# Supplementary material for: Knowledge, Attitude, Risk Perception, and Health-Related Adaptive Behavior of Primary School Children towards Climate Change: A Cross-Sectional Study in China
Source: Int J Environ Res Public Health. 2022 Nov 25;19(23):15648. doi: 10.3390/ijerph192315648 (PMC9740326; doi:10.3390/ijerph192315648)
Supplement: Supplementary file 1 [file ijerph-19-15648-s001.zip › ijerph-1949341-supplementary.pdf]

Supplementary Materials

Knowledge, Attitude, Risk Perception, and Health-Related Adaptive Behavior of Primary School Children towards Climate Change: A Cross-Sectional Study in China

Yu Wang, Xinhang Zhang, Yonghong Li, Yanxiang Liu, Bo Sun, Yan Wang, Zhirong Zhou, Lei Zheng, Linxin zhang, Xiaoyuan Yao, Yibin Cheng

Table of Contents

Table S1. Knowledge, attitude, risk perception, and adaptive behavior in the questionnaire.

Table S2. Linear correlation of variables.

Table S3. Variance inflation factor (VIF) of variables in multiple linear regression.

Table S4. Estimates in the original structural equation model.

Table S5. Estimates in the final structural equation model.

Table S1. Knowledge, attitude, risk perception, and adaptive behavior in the questionnaire.

Items (Score)	Knowledge (20)	Answers
k1 (9)	1. Select the impacts of climate change. (**multiple choice)**	①Crop decline ②Water shortage ③Local droughts/floods ④Glacier reduction ⑤Sea level rise ⑥Sea acidification ⑦Biodiversity loss ⑧Health risks ⑨Increase in extreme weather events (heatwave, cold spell, floods) ⑩None of them
k2 (6)	2. Select the mitigation methods of climate change. （**multiple choice)**	①Reduce using vehicles ②Plant trees ③Reduce/sort garbage ④Save water ⑤Save electricity ⑥Improve technology ⑦None of them
k3 (1)	3. Drinking water in time can prevent heatstroke on hot days.	①True ②False
k4 (1)	**4.** Drinking plenty of iced water can prevent heatstroke on hot days.	①True ②False
k5 (1)	5. Avoid staying outside in extreme weather events.	①True ②False
k6 (1)	6. Using public transport instead of private vehicles in extreme weather events.	①True ②False
k7 (1)	7. Adults' supervision is the most important method to prevent children from drowning.	①True ②False
**Items (Score)**	**Attitude (20)**	**Answer: ①Strongly disagree ②Disagree ③Unknown ④Agree ⑤Strongly agree**
a1 (5)	1. I am willing to know the causes and mitigations of climate change.	① ② ③ ④ ⑤
a2 (5)	2. I am willing to know the health impacts and preventive measures of climate change.	① ② ③ ④ ⑤
a3 (5)	3. I am willing to care more about my health in extreme weather events.	① ② ③ ④ ⑤
a4 (5)	4. I am willing to change my behavior to prevent diseases.	① ② ③ ④ ⑤
**Items (Score)**	**Risk perception (30)**	**Answers: ①Strongly disagree ②Disagree ③Unknown ④Agree ⑤Strongly agree**
p1 (5)	1. Climate change influences our country.	① ② ③ ④ ⑤

p2 (5)	2. Climate change is prevalent in areas where I am living.	①	②	③	④	⑤
p3 (5)	3. Climate change increases the risk of drowning, falling, or having accidental injuries.	①	②	③	④	⑤
p4 (5)	4. Climate change increases the risk of getting infectious diseases.	①	②	③	④	⑤
p5 (5)	**5.** Individuals cannot help to mitigate climate change.	①	②	③	④	⑤
p6 (5)	6. Changing my behaviors will bring health benefits.	①	②	③	④	⑤
**Items (Score)**	**Adaptive behavior (40)**	**Frequency: ①0-1 ②2-3 ③4-6 ④7-8 ⑤9-10 for every 10 times.**				
b1 (5)	1. How many days in a week do you see weather forecasts?	①0 days	②1-2 days	③3-4 days	④5-6 days	⑤7 days
b2 (5)	2. Try to get local information about preventive measures of climate change and extreme weather events.	①	②	③	④	⑤
b3 (5)	3. Wear a hat or take a sunshade when going outside on hot days.	①	②	③	④	⑤
b4 (5)	4. Wear more clothes and hats to keep warm when going outside on cold days.	①	②	③	④	⑤
b5 (5)	5. Wash hands before and after meals or toilets.	①	②	③	④	⑤
b6 (5)	**6.** Drink unboiled water or have raw foods.	①	②	③	④	⑤
b7 (5)	7. Use mosquito net or repellent when sleeping during summertime.	①	②	③	④	⑤
b8 (5)	8. Wear long-sleeved shirts or pants when visiting places with many mosquitoes.	①	②	③	④	⑤

Table S2. Linear correlation of variables.

	Gender	Grade	Knowledge	Attitude	Risk Perception	Adaptive behavior	air conditioner	heating system	Heat exposure	Cold exposure	Rainstorm exposure	PCDI levels
Gender	1.000											
Grade	0.010	1.000										
Knowledge	-0.024	0.096	1.000									
Attitude	0.046	0.064	0.300	1.000								
Risk Perception	-0.010	0.065	0.267	0.429	1.000							
Adaptive behavior	0.103	0.012	0.294	0.322	0.270	1.000						
air conditioner	0.016	-0.067	-0.071	-0.046	0.046	-0.029	1.000					
heating system	0.002	0.022	-0.023	-0.004	-0.028	-0.101	-0.139	1.000				
Heat exposure	-0.020	0.069	-0.047	-0.029	-0.067	-0.122	-0.439	0.176	1.000			
Cold exposure	0.027	-0.017	-0.121	-0.019	0.090	0.088	0.372	-0.446	-0.351	1.000		
Rainstorm exposure	-0.011	-0.013	0.129	0.107	0.085	0.139	-0.153	0.013	-0.348	0.017	1.000	
PCDI levels	-0.008	-0.014	0.182	0.102	0.035	0.113	-0.493	-0.102	0.051	-0.040	0.296	1.000

Table S3. Variance inflation factor (VIF) of variables in multiple linear regression.

Variables	VIF
Gender	1.01
Grade	1.02
Knowledge	1.21
Attitude	1.30
Risk Perception	1.29
air conditioners	1.97
heating systems	1.29
Heat exposure	1.68
Cold exposure	1.55
Rainstorm exposure	1.38
PCDI levels	1.54

Table S4. Estimates in the original structural equation model.

Variable	Items	Unstandardized estimates			Standardized estimates			Composite reliability
		Estimate	Standard error	*P* values	Estimate	Lower CI	Upper CI	
**Measurement model**								
Knowledge	k1	1.000			0.864	0.849	0.880	0.514
	k2	0.775	0.015	<0.001	0.876	0.860	0.892	
	k3	0.012	0.001	<0.001	0.100	0.077	0.122	
	k4	0.021	0.002	<0.001	0.147	0.125	0.170	
	k5	0.009	0.001	<0.001	0.100	0.078	0.123	
	k6	0.014	0.002	<0.001	0.068	0.045	0.091	
	k7	0.001	0.002	0.448	0.009	-0.014	0.032	
Attitude	a1	1.000			0.800	0.782	0.818	0.616
	a2	0.979	0.022	<0.001	0.828	0.810	0.846	
	a3	0.342	0.017	<0.001	0.251	0.228	0.274	
	a4	0.419	0.018	<0.001	0.288	0.265	0.310	
Perception	p1	1.000			0.663	0.644	0.682	0.534
	p2	0.668	0.021	<0.001	0.450	0.428	0.471	
	p3	0.824	0.025	<0.001	0.491	0.470	0.512	
	p4	0.919	0.024	<0.001	0.634	0.615	0.653	
	p5	-0.041	0.025	0.100	-0.021	-0.047	0.004	
	p6	0.662	0.022	<0.001	0.440	0.418	0.462	
Behavior	b1	1.000			0.599	0.580	0.617	0.636
	b2	0.972	0.024	<0.001	0.687	0.669	0.704	
	b3	0.769	0.022	<0.001	0.527	0.507	0.547	
	b4	0.571	0.017	<0.001	0.493	0.472	0.513	
	b5	0.520	0.017	<0.001	0.450	0.429	0.471	
	b6	0.098	0.013	<0.001	0.097	0.072	0.122	
	b7	0.350	0.021	<0.001	0.220	0.196	0.244	
	b8	0.544	0.023	<0.001	0.318	0.295	0.341	
**Structural model**								
Behavior	Knowledge	0.059	0.005	<0.001	0.169	0.139	0.199	
Behavior	Attitude	0.278	0.018	<0.001	0.240	0.212	0.268	
Behavior	Perception	0.291	0.020	<0.001	0.249	0.219	0.280	
Attitude	Knowledge	0.096	0.004	<0.001	0.322	0.298	0.346	
Perception	Knowledge	0.102	0.004	<0.001	0.343	0.318	0.369	

Note: CI, 95% confidential intervals.

Table S5. Estimates in the final structural equation model.

Variable	Items	Unstandardized estimates			Standardized estimates			Composite reliability
		Estimate	Standard error	*P* values	Estimate	Lower CI	Upper CI	
**Measurement model**								
Knowledge	k1	1.000			0.863	0.845	0.881	0.846
	k2	0.779	0.017	<0.001	0.880	0.862	0.897	
Attitude	a1	1.000			0.852	0.826	0.878	0.802
	a2	0.874	0.028	<0.001	0.787	0.762	0.812	
Perception	p1	1.000			0.662	0.644	0.681	0.664
	p2	0.669	0.022	<0.001	0.450	0.428	0.472	
	p3	0.824	0.025	<0.001	0.491	0.470	0.512	
	p4	0.919	0.024	<0.001	0.634	0.614	0.653	
	p6	0.663	0.022	<0.001	0.440	0.418	0.462	
Behavior	b1	1.000			0.612	0.593	0.630	0.668
	b2	0.980	0.024	<0.001	0.707	0.690	0.724	
	b3	0.743	0.021	<0.001	0.521	0.501	0.541	
	b4	0.543	0.016	<0.001	0.479	0.458	0.499	
	b5	0.509	0.016	<0.001	0.442	0.420	0.463	
	b8	0.501	0.022	<0.001	0.300	0.276	0.323	
**Structural model**								
Behavior	Knowledge	0.057	0.005	<0.001	0.159	0.130	0.189	
Behavior	Attitude	0.254	0.017	<0.001	0.228	0.200	0.255	
Behavior	Perception	0.312	0.020	<0.001	0.261	0.231	0.291	
Attitude	Knowledge	0.095	0.004	<0.001	0.299	0.275	0.323	
Perception	Knowledge	0.100	0.004	<0.001	0.339	0.313	0.364	

Note: CI, 95% confidential intervals.
